# Ex-ante Benefit-Cost Analysis of the Elimination of a *Glossina palpalis gambiensis* Population in the Niayes of Senegal

**DOI:** 10.1371/journal.pntd.0003112

**Published:** 2014-08-21

**Authors:** Fanny Bouyer, Momar Talla Seck, Ahmadou H. Dicko, Baba Sall, Mbargou Lo, Marc J. B. Vreysen, Eduardo Chia, Jérémy Bouyer, Abdrahmane Wane

**Affiliations:** 1 Unité Mixte de Recherche Contrôle des Maladies Animales Exotiques et Emergentes, Centre de Coopération Internationale en Recherche Agronomique pour le Développement (CIRAD), Montpellier, France; 2 Unité Mixte de Recherche Contrôle des Maladies Animales Exotiques et Emergentes, Institut national de la recherche agronomique (INRA), Montpellier, France; 3 Institut Sénégalais de Recherches Agricoles, Laboratoire National d'Elevage et de Recherches Vétérinaires, Dakar, Sénégal; 4 Direction des Services Vétérinaires, Dakar, Senegal; 5 Insect Pest Control Laboratory, Joint FAO/IAEA Programme of Nuclear Techniques in Food and Agriculture, Vienna, Austria; 6 Cirad, Unité Mixte de Recherche INRA-CIRAD Innovation, Montpellier, France; 7 Cirad, Unité Mixte de Recherche CIRAD-INRA-SupAgro Selmet, Montpellier, France; University of Edinburgh, United Kingdom

## Abstract

**Background:**

In 2005, the Government of Senegal embarked on a campaign to eliminate a *Glossina palpalis gambiensis* population from the Niayes area (∼1000 km^2^) under the umbrella of the Pan African Tsetse and Trypanosomosis Eradication Campaign (PATTEC). The project was considered an ecologically sound approach to intensify cattle production. The elimination strategy includes a suppression phase using insecticide impregnated targets and cattle, and an elimination phase using the sterile insect technique, necessary to eliminate tsetse in this area.

**Methodology/Principal Findings:**

Three main cattle farming systems were identified: a traditional system using trypanotolerant cattle and two “improved” systems using more productive cattle breeds focusing on milk and meat production. In improved farming systems herd size was 45% lower and annual cattle sales were €250 (s.d. 513) per head as compared to €74 (s.d. 38) per head in traditional farming systems (p<10^−3^). Tsetse distribution significantly impacted the occurrence of these farming systems (p = 0.001), with 34% (s.d. 4%) and 6% (s.d. 4%) of improved systems in the tsetse-free and tsetse-infested areas, respectively. We calculated the potential increases of cattle sales as a result of tsetse elimination considering two scenarios, i.e. a conservative scenario with a 2% annual replacement rate from traditional to improved systems after elimination, and a more realistic scenario with an increased replacement rate of 10% five years after elimination. The final annual increase of cattle sales was estimated at ∼€2800/km^2^ for a total cost of the elimination campaign reaching ∼€6400/km^2^.

**Conclusion/Significance:**

Despite its high cost, the benefit-cost analysis indicated that the project was highly cost-effective, with Internal Rates of Return (IRR) of 9.8% and 19.1% and payback periods of 18 and 13 years for the two scenarios, respectively. In addition to an increase in farmers' income, the benefits of tsetse elimination include a reduction of grazing pressure on the ecosystems.

## Author Summary

In 2005, the Government of Senegal embarked on a campaign to eliminate a tsetse population from the Niayes area (∼1000 km^2^) around Dakar in order to intensify cattle production. Three main cattle farming systems are present in this area: a traditional system using trypanotolerant cattle and two “improved” systems using more productive trypano-sensitive cattle breeds. Whereas the size of the herds in improved cattle farming systems is more than twice lower than in a traditional system, the annual sales per head are threefold higher. Improved systems are more than fivefold less frequent in the tsetse infested sites than in the surrounding ones, showing that the risk posed by trypanosomosis is a major constraint to the intensification and innovation processes. Based on two scenarios of shift from traditional to improved systems after tsetse elimination, the benefit-cost analysis shows that, despite its relatively high cost, the project is highly cost-effective and will allow a reduction of grazing pressure on the ecosystems.

## Introduction

**Figure 1:**
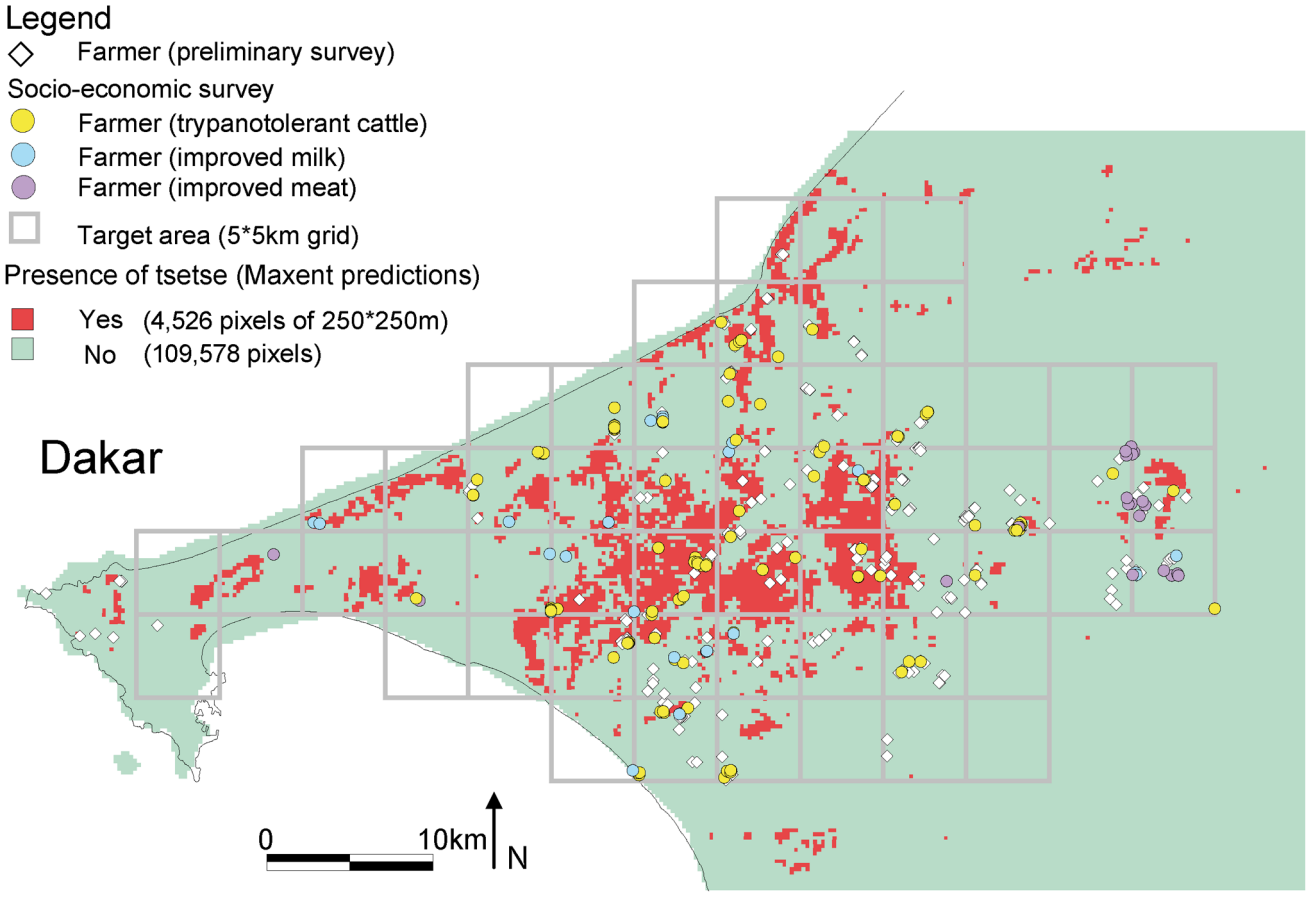
Location of the study area, between Dakar and Thiès, Senegal. The grid corresponds to 5*5 km cells that were used to design the entomological sampling strategy during the feasibility study [14]. Pixels that could harbor *Glossina palpalis gambiensis* as predicted by the Maxent model are coloured in red. White lozenges correspond to the livestock farms geo-referenced during the preliminary survey whereas circles correspond to the farms surveyed during the socio-economic survey (see text for details).

Food security and safety remains a serious concern in Africa in general and in Senegal in particular. In the last century the human population has increased tenfold in West-Africa, and is expected to triple by 2050 [1]. The expansion of current agricultural production practices will not allow feeding this increasing population which may lead to violent social crises. Senegal in West Africa faces two global challenges, namely demographic changes [2] and climatic change with especially reduced precipitation being critical as it can be associated with lower production of natural forage (ecosystem service) that is a major limit for the maintenance of traditional cattle systems in West-Africa. Moreover, overgrazing is a major cause of land degradation in Senegal [3]. The Niayes area around Dakar (Fig. 1), where the study is conducted, is partially protected from the second challenge (see below) but exposed to the first with a human population density already exceeding 150 habitants/km^2^. Fifty three percent of the Senegalese population lives in the Niayes making the competition for space severe.

In the past, the prospective increase of the milk needs in Africa (estimated to 52% over a period of ten years) [4] and the low productivity of local breeds (1–4 L/day) [5] favored the development of institutional programs for the intensification of the dairy industry in Senegal. This included embryo implants in Ndama cattle [6] and more importantly artificial insemination that first started in the area where groundnuts were the main crop but thereafter was extended to the whole country [5]. In 2010, the national production of milk was estimated at 143,124 tons for 366,200 heads of cattle, corresponding to 1.3 L/cattle/day of lactation (FAOSTAT). Milk imports have always been dominant on the Dakar market with local production only contributing 2 to 5% between 1993 and 2000 [7]. Powdered milk is 50 to 60% less expensive than local fresh milk despite the increase of taxes on this product. In 2009, the milk imports could only satisfy 60% of the total national demand (95.6 million L) [8] which was equivalent to ∼€76 million. In 2010, these imports reached ∼€96 million [9].

The Niayes area is located along the Atlantic coast of Senegal and includes four administrative districts: Dakar, Thiès, Louga and Saint-Louis. Particular meteorological and ecological characteristics of this area provide great potential for agricultural development in general and animal production (cattle, donkeys, horses, small ruminants, pigs and poultry) in particular. However, in 2004 the dairy farms of the peri-urban area of Dakar produced less than 6,000 L of milk per day. In 2005, intensified livestock production systems with exotic breeds such as Holstein, Montbéliarde, Jersey, Gir and Girolando, and cross-breeds between this breeds and local cattle were only found on 1% of the farms. Mean daily milk production was still limited to 6.9 L (s.d. 3) despite much higher genetic potential of these exotic breeds and the use of large amounts of inputs (food concentrates, drugs, …) [10]. From 1984 to 1993 these farms received government support that included training, animal health care and feed ingredients. Despite this support, farmers were still disorganized in 2005 in terms of milk distribution and inputs. In 2008, a project called “La Grande offensive agricole pour la nourriture et l'abondance (GOANA)” (http://www.gouv.sn/IMG/article_PDF/article_777.pdf) was launched which included a component of artificial insemination of local breeds with exotic dairy breeds and by December 2011, more than 91,000 cattle had been inseminated.

In view of its proximity to the Atlantic Ocean, the Niayes area is a particular eco-region that is more resilient to climate change as compared to other regions in Senegal e.g. the area only experienced a reduction of 150 mm in annual precipitation the last 20 years compared to 200 mm of precipitation in the rest of Senegal. Unfortunately, this microclimate also favours the presence of *Glossina palpalis gambiensis* Vanderplank, a riverine tsetse species. Tsetse flies (Diptera: Glossinidae) are the vectors of human African trypanosomosis (HAT) and African animal trypanosomosis (AAT), the former a major neglected human tropical disease, and the latter considered among the greatest constraints to improved livestock production in sub-Saharan Africa [11]. Most domestic animals are susceptible to AAT which was until recently still highly prevalent in the Niayes area [12]. It was a major pathological problem especially for cattle crossed with exotic breeds and Gobra zebus which are very susceptible to trypanosomes.

The sustainable removal of the vector, the tsetse fly, would be the most efficient way of managing AAT [13]. In 2001, an African Union initiative called the Pan African Tsetse and Trypanosomosis Eradication Campaign (PATTEC) was launched following an historic decision by the African Heads of State and Government in Lome, Togo, July 2000. In 2005, the Senegalese Government joined this campaign, starting a tsetse control campaign that aimed at the elimination (elimination is here considered as local eradication) of *G. p. gambiensis* from the Niayes area (Fig. 1). The program is implemented by the Government of Senegal (Direction of Veterinary Services (DSV) and the Senegal Institute for Agricultural Research (ISRA)) and technically and financially supported by the International Atomic Energy Agency (IAEA), the Food and Agriculture Organization of the United Nations (FAO), the Centre de Coopération Internationale en Recherche Agronomique pour le Développement (CIRAD) and the USA through the Peaceful Uses Initiative (PUI) (www.fao.org/news/story/en/item/211898/icode/). During the feasibility study of this project the limits of the *G. p. gambiensis* distribution were determined to be within a 1,000 km^2^ area (Fig. 1) and it was demonstrated that this population was completely isolated from the main tsetse belt in the south-eastern part of Senegal [14], [15]. Therefore, the Government of Senegal selected a strategy of elimination following area-wide integrated pest management (AW-IPM) principles [16] to create a sustainable zone free of *G. p. gambiensis* in the Niayes. The strategy combined insecticide-treated targets and cattle for initial fly suppression [17] with the aerial release of sterile male flies as the final elimination component [18]. The study area was divided in four operational blocks that are being treated sequentially. At the time of writing, 20% of the project area was already cleared of *G. p. gambiensis* (no capture of wild flies during 18 months in the monitoring traps) and the apparent density of the fly population had been reduced with 99% in an additional 40% of the project zone.

This project constitutes a major governmental intervention that will have a great positive impact on the Niayes agro-ecosystem. The goal of this paper is to present an ex ante benefit-cost analysis of this project which includes an SIT component that is considered by many a costly control tactic [19].

## Methods

### Evaluation of the benefits

#### Sampling strategy and socio-economic survey

During the feasibility phase of the elimination project, field staff of the Ministry of Livestock conducted preliminary surveys in the target area and geo-referenced ∼50% of the livestock farmers in their respective districts, and classed the cattle herds in three size categories (<20, 20–100, >100). The 513 geo-referenced farms were used to develop a stratified sampling strategy based on cattle herd size and 192 farms were selected as a representative sample for each group (see Results section). These farms were surveyed from July to November 2010 using a carefully developed questionnaire. The final datasets of 186 farmers were considered accurate and these were used for the statistical analyses.

The questionnaire was designed to capture instantaneous or recent data (between the two last rainy seasons) and contained mainly closed questions that allowed the collection of quantitative data sets. The first section of the questionnaire dealt with the composition of households (sociological structure, main occupations of the head of the family), the second with equipment and farming resources, and the third with the composition of the livestock and their productivity. Detailed data were collected on cattle including herd structure (numbers per sex and age) and their productivity. Sales data were collected by cattle species for meat and milk production systems. The health constrains were prioritized by the farmers and the treatments were listed by species.

#### Types of livestock production systems

Different farming systems were identified based on herd composition and cattle breeds. Tsetse presence/absence was assessed using habitat suitability as predicted by a Maxent model [20] that was developed using tsetse presence data obtained during the feasibility study of the tsetse elimination project [14] and environmental data derived from Landsat and MODIS data. A threshold of 0.13, corresponding to a sensitivity of 0.96 and a specificity of 0.57 was used to discriminate tsetse infested pixels from non-infested pixels [21].

#### Statistical analyses

The impact of farming system on cattle productivity (e.g. herd size, milk production) and economic variables (e.g. animal sales price, annual sales) was assessed using an analysis of variance followed by Student's t tests between the groups. Binomial generalized linear models were used to compare cattle mortality and birth rates within the farming systems.

The main indicator used to calculate benefits in this study was “cattle sales” corresponding to the total cash income for livestock producers coming from animal products (milk and meat). For statistical comparisons between farming systems, the sales of live animals were divided by the total number of cattle present in the farm, and the milk sales were divided by the number of producing females. The data used in this analysis are presented in Supporting information S1.

### Evaluation of the costs

**Figure 2:**
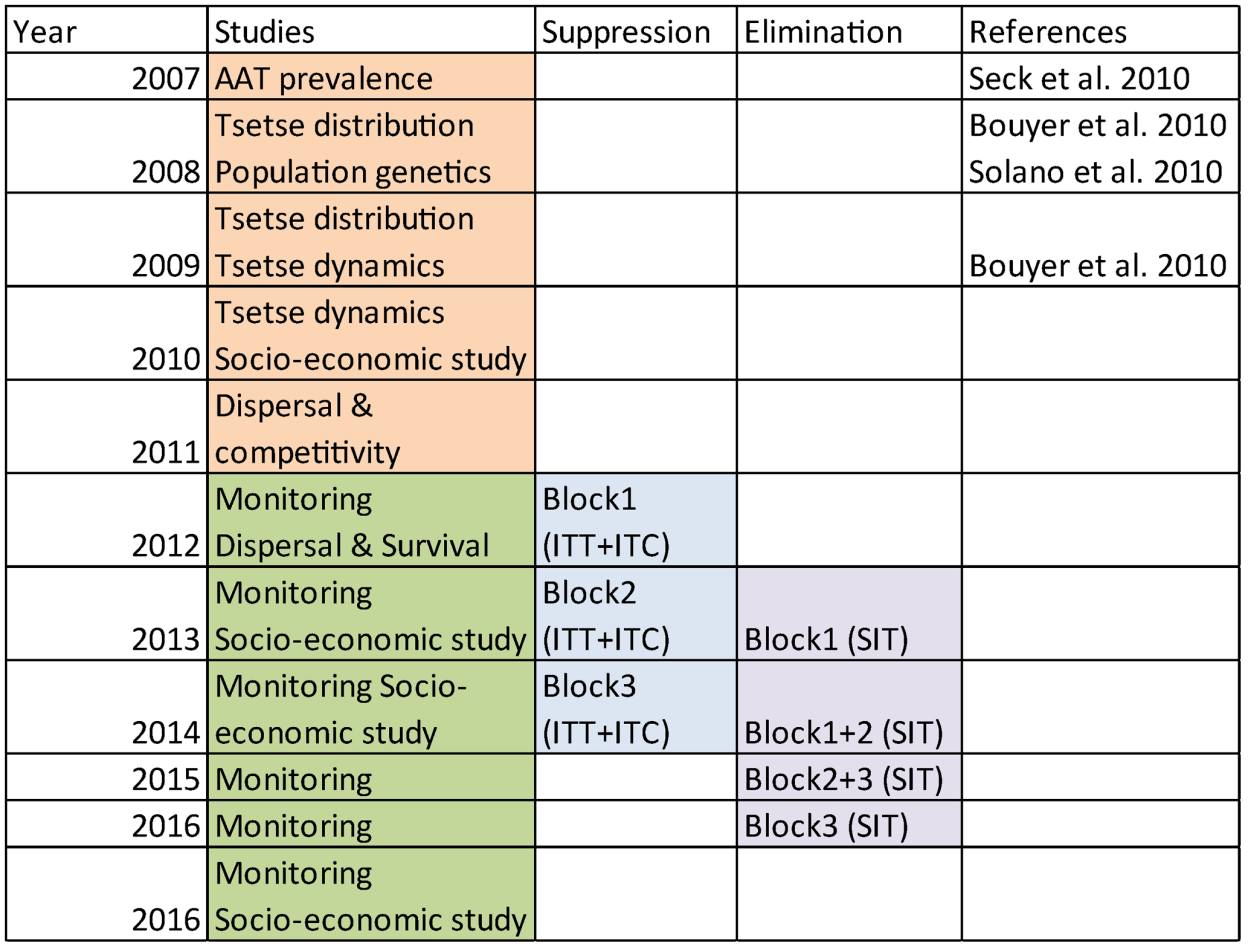
Time table of the tsetse elimination project in the Niayes. This table was used as a basis for the cost calculations.

Since the project has not been completed at the time of writing, cost estimates were based on real expenditures until December 2013, and anticipated expenditures until December 2016. The general economic framework proposed by [19] was used to class the costs into three main areas: studies, field costs and administration. Administration costs from 2007 to 2011 were included in the cost of studies because the operational phase of the tsetse project (control) started only in January 2012 (fig. 2). The field costs were further subdivided into a core component (traps, pour on, sterile flies, cost of aerial release) and other expenditures such as vehicle running costs, salaries and field allowances.

### Evaluation of the main financial ratios and scenarios

In this study, we assumed no increase in cattle numbers. The ex-post socio-economic survey conducted on Unguja Island, Zanzibar after the elimination of a *Glossina austeni* population showed an estimated initial replacement rate of 2% per year of traditional to improved livestock breeds [22]. In the absence of any other study of this type, we used this replacement rate to estimate the potential benefits of the elimination campaign in the Niayes. This was considered a very conservative scenario as innovation sociology dictates that the introduction dynamics of exotic or more productive breeds follows an S curve, i.e. the rate of adoption should increase after the first period spearheaded by the early innovators [23]. However, we also tested a second more realistic scenario where the replacement rate was set at 10% following an initial period of 5 years with a replacement rate of 2%.

The monetary assessment of the benefits and costs of the benefit-cost analysis was based on the calculation of the Payback Period (PP), the Net Present Values (NPV), the Internal Rate of Return (IRR) and the benefit-cost ratios.

The PP refers to the period of time required for the operational products minus the operational expenses to recover the funds placed in an investment. The NPV is the monetary surplus at the end of a project after refunding the invested capital on the total period of the project and the accounts balance initially invested according to the selected discount rate, 
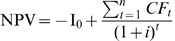
with 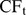 the annual cash flow, 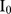 the initial investment and 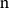 the duration of the project. The IRR corresponds to the discount rate I for which the NPV of a project is null, 
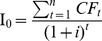
.

We selected two main hypotheses for the analysis:

- Discount rates of 5% and 10%, that have been used for similar animal health projects [19], [24]. The “time preference” concept is based on the fact that getting an income today is more valuable than getting the same income in the future; the discount rate thus allows accounting for the present value of financial flows that will take place in the future.
- A period of 30 years to measure the full effect of the elimination since it is anticipated that the project will take 10 years to complete [19].

In our study, prices were calculated based on a conversion rate of 655.956 FCFA for one Euro and constant 2013 prices were applied throughout the projection.

### Ethics statement

All farmers provided informed consent before filling the forms. The consents were oral to ensure equal treatment of the subjects, since a large part of the farmers were illiterate. The survey was approved by the General Director of Vet Services and conducted by the agents of Veterinary Services, in charge of animal health in Senegal.

## Results

### Description of the farming systems

The sample comprised 8,488 cattle of which 5.4% were dairy cattle breeds. During preliminary surveys 44% (226), 52% (267) and 4% (20) of the farmers were classed in the cattle herd categories <20, 20–100, and >100 respectively. From these, 39, 131 and 16 farms took part in the socio-economic survey respectively, and they had a mean herd size of 13, 44 and 133 animals respectively. Based upon this, the number of cattle in the farms surveyed during the preliminary survey was estimated at ∼44,111 animals which can be extrapolated to 80,000–90,000 resident cattle in the target area of the Niayes.

**Figure 3:**
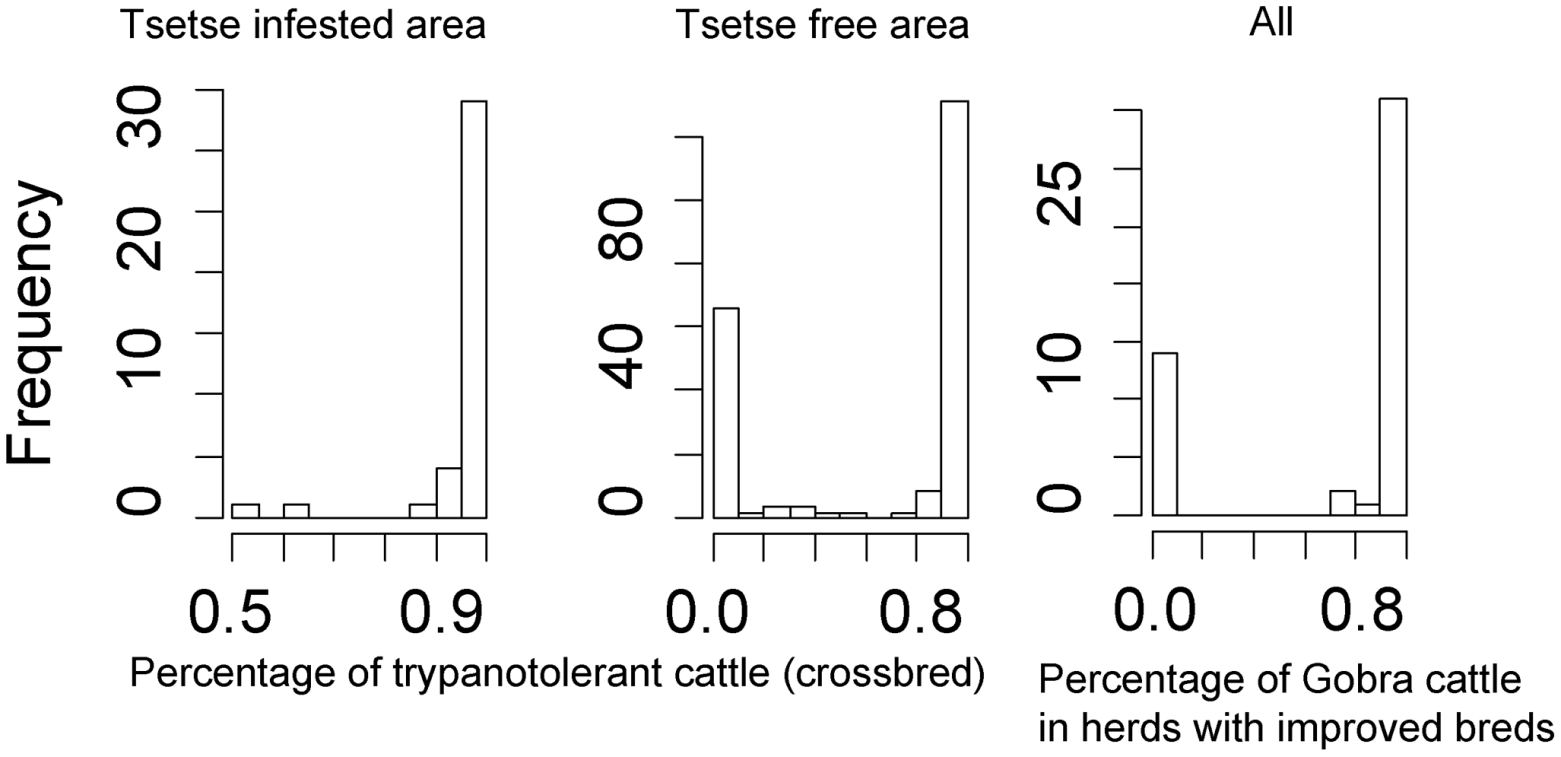
Frequencies of cattle breeds in relation to tsetse presence and farming system. The frequency of trypanotolerant cattle can be associated with two dominant groups, one based mainly on trypanotolerant cattle, and the second based on improved breeds, present almost only in the tsetse free area. The latter can be subdivided into two sub-groups based on the frequency of Gobra zebu cattle, which is the main breed for meat production in the area: an improved livestock keeping system targeting milk production, and a second one targeting meat production.

Three clusters of livestock keeping systems were identified. The first one was traditional and based mainly on trypanotolerant cattle (more than 70%) called “Djakoré” in the study area. These cattle are a cross between “Gobra”, the main zebu breed originating from northern Senegal, and “Ndama”, a trypanotolerant breed originating from the main tsetse belt in south eastern Senegal (fig. 3). The two other livestock keeping systems used more productive breeds and were composed of meat producing farms with mainly Gobra cattle (>70%), and farms targeting milk production where less than 10% of the cattle were Gobra (fig. 3).

There was a strong impact of tsetse presence (as assessed using the Maxent model) and the frequency of the type of farming system (X-squared = 10.1748, df = 1, p-value = 0.001) with 34% (s.d. 4%) of farmers owning improved breeds in the tsetse-free pixels compared to 6% (s.d. 4%) only in the tsetse-infested pixels.

The farming systems are henceforth denoted trypanotolerant, improved meat and improved milk. The Fulani are an ancestral ethnic group of cattle breeders who were the predominant group in the sample (82%) especially in the traditional trypanotolerant system (90%). They were dominant in the improved meat (69%) but not in the improved milk (36%) farming system where the Wolof ethnic group was the most frequent (43%). The other ethnic groups were the Sérères, the Toucouleurs and the Lebous. The Toucouleurs together with the Fulani constitute the larger ethnic group of the Al Pulaar who were dominant even in the improved milk farming system (50%). In general, 57% of the farmers considered AAT as the main animal health problem with a marked difference between the tsetse-free (49%) and -infested area (92%). All farmers of the improved milk farming system that were located in the tsetse-infested area considered the AAT as the major animal health problem with ticks coming second. Despite increased competition for space in the main cities, there was no clustering of improved milk farming systems in Dakar or Thiès, where tsetse also occur (fig. 1).

### Cattle sales

**Figure 4:**
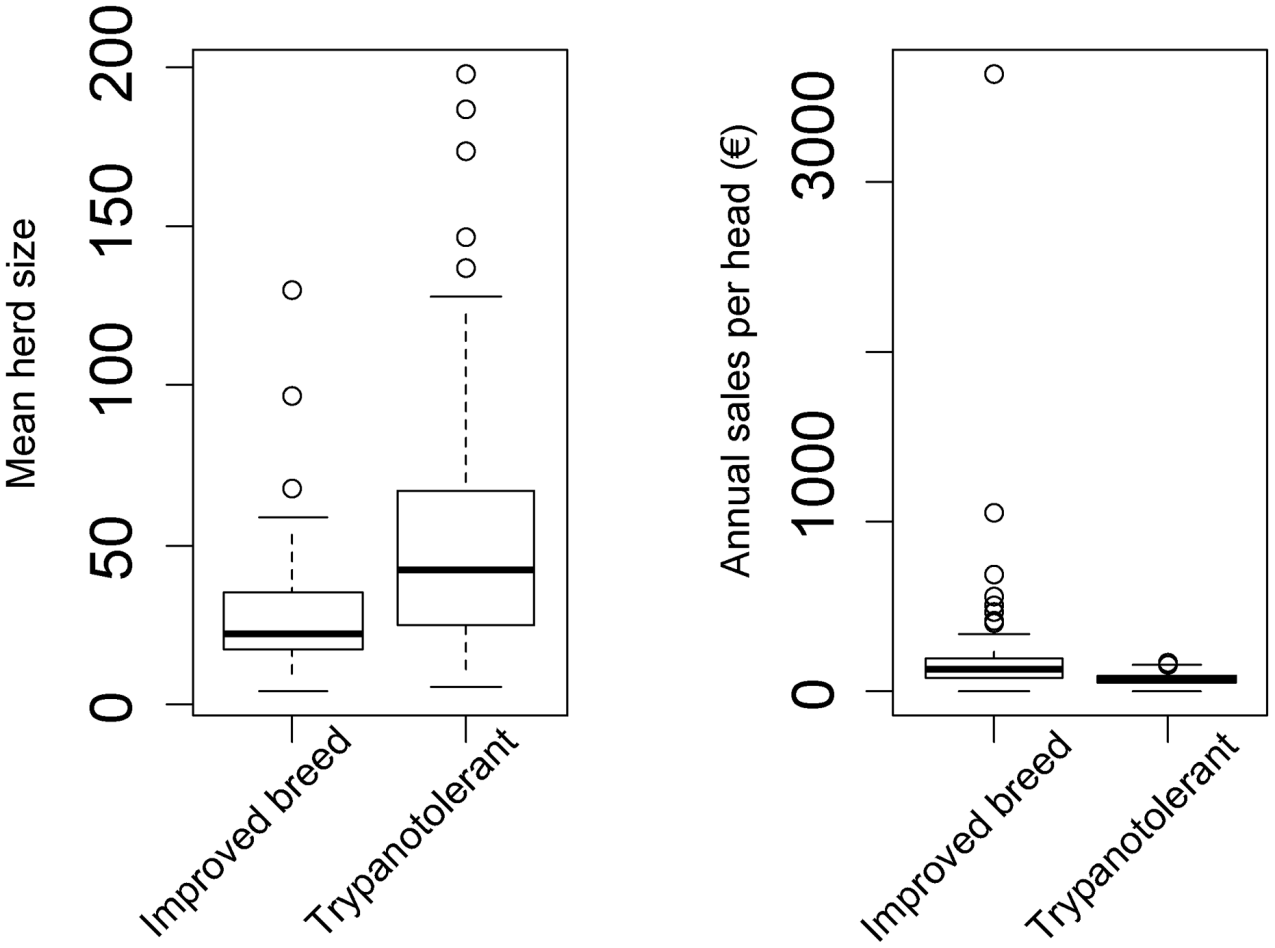
Boxplots of cattle herd size and annual sales of improved and trypanotolerant cattle. Annual sales correspond to the total sales divided by the number of cattle present in the farm.

There was a significant effect of type of farming system on the annual cattle sales (p<10^−3^, fig. 4). Despite the different production schemes in the improved milk and improved meat farming systems, the annual cattle sales were similar in the two systems (t = −0.9577, df = 50.999, p-value = 0.3427) averaging € 250 (s.d. 513) per head which was more than 3 times higher than in the trypanotolerant system (€74, s.d. 38). The difference was significant for both the improved milk (t = −1.815, df = 13.088, p-value = 0.046) and improved meat (t = −2.164, df = 38.086, p-value = 0.018) farming systems. These increased sales were obtained through more animals being sold and higher prices obtained per head in the improved meat farming system and through increased milk production in the improved milk farming system (table 1). The price of the milk was similar for all farming systems, i.e. €0.73 per L (s.d. 0.09).

**Table 1:** Description details of the three farming systems encountered in the Niayes.

Farming system	Trypanotolerant	Improved meat	Improved milk
Percentage of the sample	71.5% (n = 133)	21.0% (n = 39)	7.5% (n = 14)
Percentage of Djakoré cattle	98.4%(s.d. 3.1%)	**0%(s.d.0%)** ***	**13% (s.d. 21.5%)** ***
Percentage of Gobra cattle	0.1%(s.d. 0.7%)	**98.2%(s.d. 5.6%)** ***	**0.5%(s.d. 2.1%)** ***
Percentage of exotic cattle	1.5%(s.d. 3.0%)	1.8%(s.d. 5.7%)	**65.0%(s.d. 40.5%)** ***
Crop surface (ha)	2.0(s.d. 2.4)	2.1(s.d. 5.4)	**7(s.d. 10.7)** *
Grazing area owned (ha)	0.03(s.d. 0.35)	0.85(s.d. 4.82)	**2(s.d. 3.86)** *
Milk sold per producing female (L)	157 (s.d. 110)	**110 (s.d. 64)** ***	339 (s.d. 463)°
Number of cattle sold	3.5 (s.d. 3.6)	**10.5 (s.d. 20.7)** *	3.6 (s.d. 5.5)
Price per head (€)	266 (s.d. 178)	**440 (s.d. 163)** ***	232 (s.d. 190)
Herd size	52 (s.d. 38)	**28 (s.d. 18)** ***	**31 (s.d. 32)** *
Calving rate	0.62 (s.d. 0.16)	**0.47 (s.d. 0.19)** ***	0.68 (s.d. 0.19)
Adult mortality rate (>2 years)	0.04 (s.d. 0.12)	**0.05 (s.d. 0.10)** **	**0.01 (s.d. 0.05)** ***
Calf mortality rate (<2 years)	0.07 (s.d. 0.13)	**0.10 (s.d. 0.19)** *	0.04 (s.d. 0.16)
Yearly cost of trypanocides per head per year	0.55 (s.d. 0.36)	**0.37 (s.d. 0.27)** *	**1.27 (s.d. 1.29)** *

The statistics presented in this table correspond to 186 farms for which all data were available. Statistical tests presented in the table correspond to a comparison of the improved farming systems with the trypanotolerant one. All the production rates are annual and the figures per farm (unless specified). Bold numbers indicate significant differences,

° p<0.1,

* p<0.05,

** p<0.01,

*** p<0.001).

The average herd size in the improved milk and meat farming systems was similar (28.6, s.d. 25.5) but was on average 45% smaller than in the trypanotolerant farming system (52.4, s.d. 37.5).

Concerning other cattle production parameters (table 1), the improved meat farming system was similar to the trypanotolerant system in terms of herd management (free grazing in communal land, cropped areas, no employees) but experienced higher cattle mortality and lower calving rates as compared to the trypanotolerant system, probably because of the higher sensitivity to trypanosomosis of Gobra cattle. The improved milk farming system showed better cattle production parameters, probably because much more inputs were used in this system, as demonstrated by the higher yearly cost of trypanocidal drugs. The declared mean amount of money spent on trypanocidal drugs was generally very low, but significantly higher in the improved milk than in the trypanotolerant system indicating some degree of exposure to trypanosomes even if most of them where located adjacent to the tsetse-infested pixels. They were significantly lower in the improved meat system than in the two other systems.

### Evaluation of the costs

**Figure 5:**
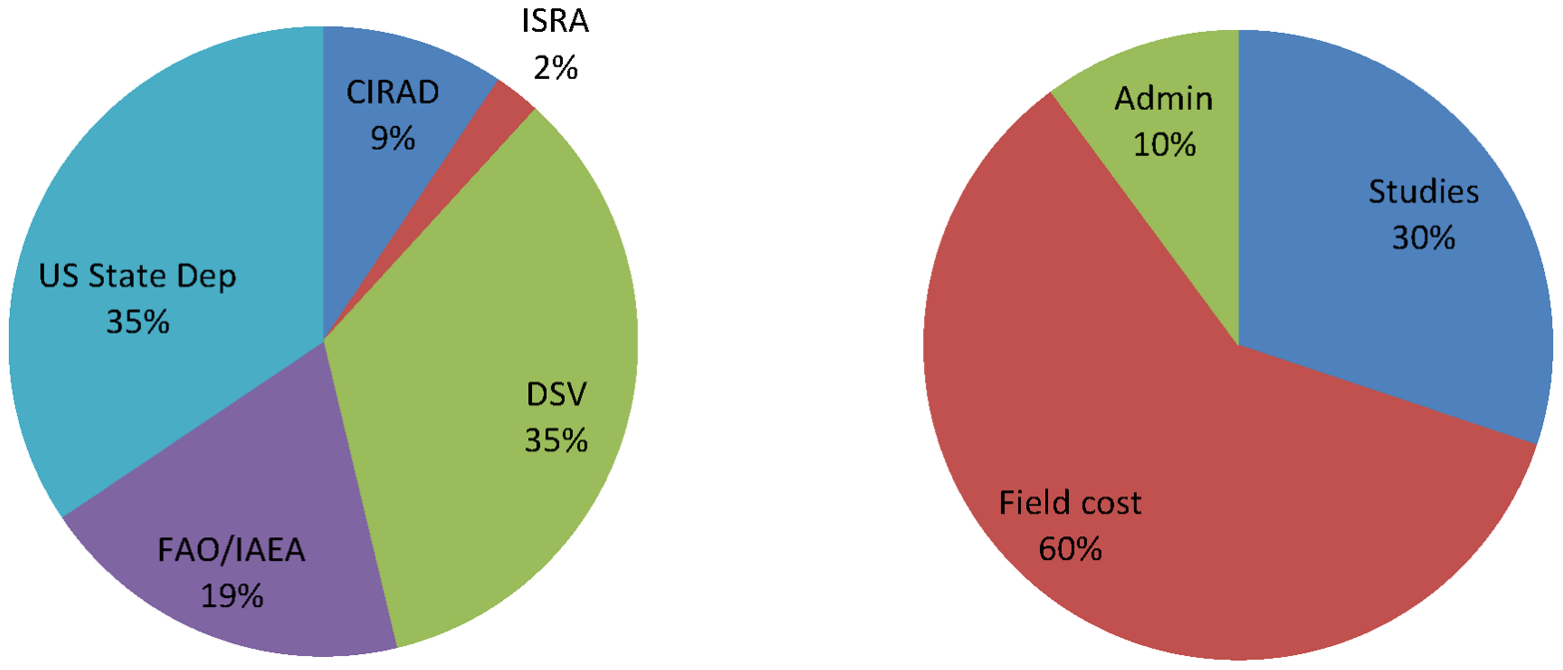
Distribution of the costs by partner (left) and component (right).

The total cost of the tsetse elimination project was estimated at € 6.4 million (table 2) contributed by the Ministry of Livestock of the Government of Senegal, the ISRA, the US Department of State, the FAO, the IAEA and the CIRAD. The total contribution from Senegal reached 37% of the total cost and the breakdown of the other contributions is presented in fig. 5.

**Table 2:** Breakdown of total costs of the tsetse elimination project in the Niayes area.

Component of total costs	Absolute value (Millions euros)	Percentage of total costs
**Core components** (traps, pour ons, sterile males, flying time)	**1.90**	**29**
**Other field costs** (vehicles, vehicle running costs, field indemnities)	**1.97**	**30**
**Entomological studies** (distribution, population genetics & dynamics, competitiveness, survival, dispersal, monitoring)	**1.44**	**22**
**Other studies** (parasitological, socio-economic, environmental)	**0.51**	**8**
**Administration (salaries, expert missions, meetings)**	**0.66**	**10**
**Total**	**6.47**	**100**
**Ratio of overheads/field costs**	**0.7**	**-**
**Field costs/total costs (%)**	**-**	**59**

The core component of the field costs (table 3) corresponded to the insecticide impregnated monoconical traps (n = ∼3600 with purchase value of each €3), the insecticides to treat ∼25,000 cattle at monthly intervals during the suppression phase (total of 6 times) (pour on cost of €0.30/treatment) and the aerial release of sterile males (∼2.8 million sterile male pupae purchased from the CIRDES and the Institute of Zoology, Slovak Academy of Sciences, Bratislava, Slovakia at €0.15 and €0.17/pupae respectively, €0.04 transport cost/pupae from Bobo Dioulasso, Burkina Faso or Bratislava, Slovakia to Dakar, Senegal and 4,000 hours of flying time with gyrocopters to disperse the sterile flies at €320 per hour including airport costs). These costs corresponded to the following treatment schedules: ∼17 insecticide impregnated traps/km^2^ of suitable habitat (total surface area of 231 km^2^ according to the Maxent predictions) with a 50% replacement rate during the suppression phase of one year; 2.5 cattle/km^2^ treated 6 times with pour-on at monthly intervals; 27 sterile flies released per km^2^/week i.e. 117 per km^2^ of suitable habitat/week (2 releases by week) with a swath of 500 m between release lines.

**Table 3:** Breakdown of core components costs of the tsetse elimination project in the Niayes area.

Elements of Core components	Absolute value (k euros)	Percentage of core components
Traps (3.6/km^2^)	**10.8**	**0.6**
Pour ons (2.5/km2)	**45**	**2.4**
Sterile males (27/km^2^/week)	**561.6**	**29.6**
Flying time (4,000 hours)	**1,280**	**67.5**
**Total**	**1,897.4**	**100**
**SIT costs/Core components (%)**	**-**	**97.1**

Other field components included three 4*4 vehicles, their running costs (fuel, spare parts) and the field allowances for the field staff who implemented the suppression and the elimination phase from 2012 to 2016 (fig. 2).

Entomological studies included the demarcation of the target population (2007–2009) [14], the confirmation that the Niayes population was completely isolated from the remainder of the tsetse belt in South East Senegal through a population genetics study (2008) [15], the monitoring of the population dynamics of the fly population (2009 to 2011) (including apparent densities as revealed by trap catches, trypanosome infection rates in the flies, physiological age distribution, and natural abortion rates), the assessment of the survival, competitiveness and dispersal of sterile males in the different ecosystems of the target area during trial releases of more than 240,000 sterile male *G. p. gambiensis*. The costs of the SIT component of the entomological study (competitiveness, survival and dispersal of sterile males) represented 46% of the total entomological study costs.

Other studies included a parasitological base line data collection in the entire target area (2007) [12], a monitoring of the AAT incidence as of 2009, an environmental monitoring using various ecological indicators (as of 2010) [25], a socio-economic study including the ex-ante transversal study presented here, an assessment of farmer innovation trajectories and an ex-post transversal study planned in 2016.

Finally, administrative costs included monthly coordination meetings, expert missions, external reviews and salaries of the staff and advisors. The staff of the project was composed of 2 doctors of veterinary medicine (35% of their time), 3 agricultural engineers (50%) and 7 technical staff (50%) of the DSV, 1 senior researcher (50%), 1 junior researcher (20%) and 3 entomological technicians (100%) of the ISRA and 1 senior researcher (50%) of the CIRAD. There were in addition MSc (6) and PhD students (3) involved in various project activities.

### Benefit-cost ratio of the project

**Figure 6:**
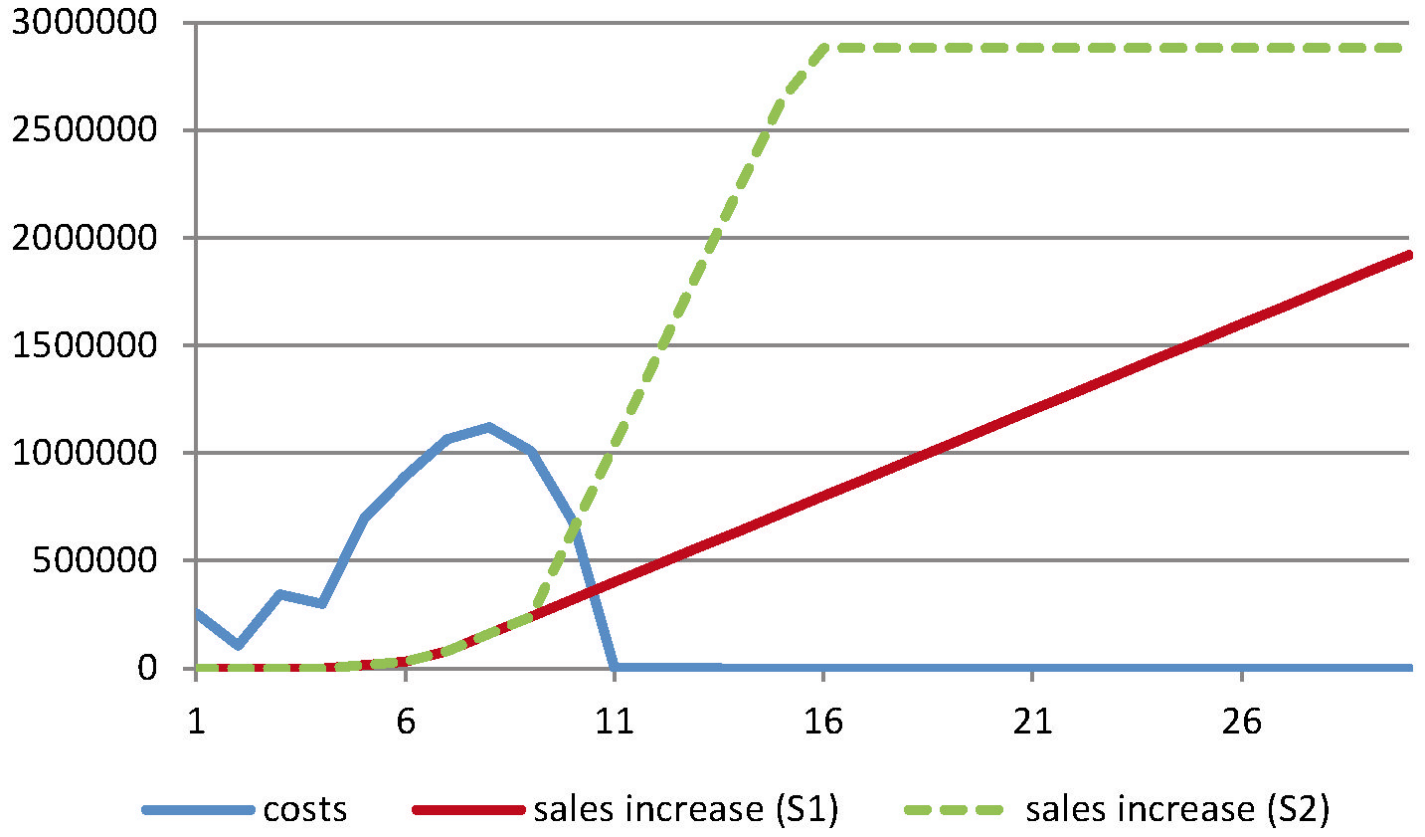
Comparison of the total costs of the project and increase in global cattle sales. The figures (Euros) concern the Niayes area over a period of 30 years after the beginning of the project. Cattle sales include meat and milk sales. S1 corresponds to the scenario with a constant 2% annual replacement rate from traditional trypanotolerant farming systems to improved farming systems, and S2 to the scenario with an accelerated replacement rate according to the sociology of innovation (see text for details).

The first effect of the project on the sales of meat and milk was seen in year six of project implementation when elimination of the *G. p. gambiensis* population was obtained in the first block (20% of the area). In year seven and eight, the total area of elimination was assumed to reach 60% and 100% of the total target area respectively (Fig. 6). Except for scenario 1 that assumes a 2% annual replacement rate with a discounting rate of 10%, the project has a positive NPV and an IRR higher than average interest rates for financing the project (table 4). The payback period would be 18 years for the first scenario and 13 years for the more realistic scenario 2 (corresponding to 2020). The benefit-cost ratios ranged from 0.98 to 4.26 depending on the discount rates and scenarios (table 4).

**Table 4:** Main financial indicators of the tsetse elimination project.

	Pay-back period	Internal Rate of Return (IRR)	Net Present Values (NPV)	Benefit-cost ratio
Scenario 1	Year 2025	9.8 per cent	3,774,500 €	1.81
Discount rate of 5 percent				
Scenario 2	Year 2020	19.1 per cent	15,240,855 €	4.26
Discount rate of 5 percent				
Scenario 1	Year 2025	9.8 per cent	−85,874 €	0.98
Discount rate of 10 percent				
Scenario 2	Year 2020	19.1 per cent	4,838,266 €	2.39
Discount rate of 10 percent				

Projections correspond to a total project duration of 30 years, two scenarios of innovation and two discount rates. S1 corresponds to the scenario with a constant 2% annual replacement rate from traditional trypanotolerant farming systems to improved farming systems, and S2 to a scenario with an accelerated replacement rate according to the sociology of innovation (see text for details).

Depending on the scenario, 100% of the farmers will have shifted from trypanotolerant cattle to more productive breeds in 2048 (scenario 1) or in 2022 (scenario 2) and the annual increase of cattle sales will reach more than €2.8 million, i.e. a 54% increase in total sales. Over the same period, the total cattle population will be reduced by 45%.

## Discussion

The total cost of the project was estimated at ∼€6400/km^2^. This is much higher than the estimated costs calculated by Shaw et al. [19] in a hypothetical programme that removed an isolated population of *G. fuscipes fuscipes* in a 10,000 km^2^ area of Uganda, i.e. €353 for ITT, €161 for ITC, and €726–998 for SIT in addition to the other techniques. The cost of a programme that combines the three techniques would thus be €1268–2784/km^2^, despite potential administrative savings when integrating ITT and ITC in the same project. Our estimation for the Niayes programme is thus 2.3 to 5 times higher than these predictions, which might be attributed to the relatively small target area which prevents economies of scale, like producing the sterile flies in Senegal instead of procuring them abroad. Considering that ∼50% of the administrative and “other field” costs were attributable to SIT, the addition of an SIT component corresponded to ∼59% of the global costs i.e. ∼€3800/km^2^. This is also much higher than the predictions of Feldmann 2004 for riverine tsetse in West Africa (€185–222/km^2^ for a release density of 10 sterile males/km^2^) or even for savannah species (€593/km^2^ for release rates of 50–100 sterile males/km^2^) [26]. Our estimate is also higher than that of Brandl 1988, who predicted a cost of ∼€472/km^2^ for ITT and ∼€690/km^2^ for SIT (estimated from [27] after taking into account inflation), although in the case of the Sidéradougou campaign, sterile males were released from the ground which is less costly when the target areas are relatively small [28]. Finally, our estimate is closer to what was found for the cost of the Zanzibar program (€2,600/km^2^ for a release density of 50–300 sterile males/km^2^) [29]. The usefulness of adding an SIT component to reach complete elimination has been debated in the literature recently [19], [30], [31] and this debate is beyond the scope of this study. In Senegal however, the suppression phase was very efficient (90–99% reduction of the original fly population density) but was followed by a stagnation (levelling off) of the tsetse apparent density which later increased again despite the maintenance of the targets: only the sterile males allowed complete elimination [32]. The same results against the same sub-species were also obtained on the Loos Islands in Guinea [33] where the target populations were also completely isolated [34]. These observations can mainly be attributed to (1) the metapopulation structure of the target population, making it impossible to deploy insecticide-impregnated targets in all suitable habitat patches [35], (2) to the density-dependent efficacy of insecticide-impregnated targets, (3) to the invers density-dependent efficacy of SIT (as the ratio of sterile to wild males will increase with population reduction and each generation), and (4) to the efficiency of the sterile males to find the last remaining virgin wild females [18]. In Senegal, the use of a maximum entropy modeling to better target insecticide-impregnated targets in suitable habitat patches and adapt the release densities of sterile males to the area of suitable habitats reduced the costs by ∼€44/km^2^ for the ITT component and by ∼€590/km^2^ for the SIT component [32].

The predicted threefold increase in cattle sales (milk and meat) as a result of the removal of the *G. p. gambiensis* population was however so significant that the programme remains highly cost-effective. Although there are some tsetse infested areas in Africa where zebu or even dairy cattle breeds could be successfully introduced, there is no reason to keep trypanotolerant livestock in the absence of trypanosomoses in our study area, where the Gobra breed has a better productivity than the Djakoré breed (table 1). The average body weights at sale were for example 188.5 kg (s.d. 80.0 kg) and 141.4 kg (s.d. 102.4 kg) for Gobra and Djakoré cattle, respectively (p<0.05). The assumed 2% annual replacement rate towards more productive cattle is thus quite conservative. Case studies of socio-technical networks are presently being conducted in the Niayes and have so far revealed that farmers consider trypanotolerant livestock as more resilient but zebu and exotic cattle as more productive and beautiful. Assuming a more realistic annual replacement rate, all traditional farming systems would have been replaced by more productive ones by 2022. In a recent study conducted in East Africa, maximal benefits (increase of income) associated with the removal of trypanosomoses were estimated at on average €2390/km^2^ over a 20 years period (ranging from €360/km^2^ to €7240/km^2^ for a 10% discount) [36]. Our estimates of the sales increases over a 20 years period would be €13,700/km^2^ and €36,300/km^2^ for scenarios 1 and 2 respectively, but this does not account for potential increases of production costs. Moreover, previous models [36] anticipated an increase of cattle density which is difficult to compare with the peri-urban situation around Dakar, where increasing land pressure allows for intensification only. The absence of clustering of improved milk farming systems in the main cities (fig. 1), where competition for space is even more important, is probably due to the presence of tsetse populations that survived in the many mango and citrus tree plantations along the Dakar-Thiès axis. These plantations became the preferred habitat of *G. p. gambiensis* in this area as the natural forest vegetation disappeared [14].

Overall, the benefit-cost ratios estimated in our study ranged from 1∶1 to 4∶1 and are thus lower than the estimations of Kristjanson et al. 1999 who used very favorable assumptions in their models (discount rate of 5%, adoption rates of the innovation of 30% and a 13.3% yearly increase of the herd size) [24]. Although the area affected by the control activities is relatively small and the demand much higher than the offer, it is possible that increased supply might result in a slight price reduction and a subsequent consumer surplus, offsetting some reduction in the producer's surplus due to lower prices. Modelling this was however outside the scope of the study.

Although the meat and milk farming systems present similar total sales, the replacement of the traditional farming system with improved meat systems is much more likely to occur than with improved milk production in view that the trypanotolerant and improved meat farming systems have similar practices and organization in addition to the fact that the Gobra breed is much more productive than the Djakoré in our study area (table 1). That is why the Conseil National de Concertation et de Coopération des Ruraux of Senegal considers that farmers are much more attracted to this type of intensification dynamics [37]. Like in Burkina Faso, farmers adapt the level of trypanotolerance of their herds to the risk of AAT and make a trade-off between resilience and productivity [38], [39]: more productive breeds will thus be selected with the reduction of this risk. However, they tend to maintain a similar farming system, thus selecting breeds than can adapt to other constraints associated to traditional systems, like natural grazing. The Gobra cattle originate from northern Senegal and are in comparison to dairy breeds much more resilient to quantitative and qualitative changes in food availability. Improving milk production would thus necessitate further commitments from the Government of Senegal beyond artificial insemination or distribution of cattle feed, and extended to the whole dairy production chain including marketing. Moreover, shifting to improved farming systems would require a global change of cattle management practices and social norms of the socio-technical regime. Actually, changing one practice only (here substituting the cattle breeds by exotic ones) would be doomed to fail since herd management practices, use of veterinary services, food supply and products sales must change as well. This evolution must be reinforced based on participatory approaches (collective management). The study of socio-technical transition pathways shows that the diffusion of an innovation does not correspond to an isolated substitution of practice but to a new configuration of the socio-technical regime: it means new norms, new relations between actors and the simultaneous change of associated practices [40]. The problem of forage production is of particular importance, since dairy farmers are competing for land with farmers that produce cereals and vegetables for human consumption. In the absence of complementary measures, the final proportion of improved milk systems will probably represent only 26% of the farms.

In this study, some factors might have contributed to underestimate the benefits of the programme. First, only cattle were considered whereas many other livestock species, including horses and sheep are trypano-sensitive and they will therefore also benefit from the removal of the tsetse fly and the trypanosomosis problem. We have assumed this simplification in the benefit-cost analysis because previous studies revealed that the trypanosome prevalence was much higher in cattle [12] and farmers considered AAT as the most important pathological constraint mainly for cattle. Second, even if the current improved farming systems are mainly outside the tsetse infested pixels, the cattle can still be exposed to tsetse during grazing (especially for improved meat farms that practice free grazing) [39], or by active dispersal of tsetse up to 3–4 km from the suitable habitats especially during the rainy season [41]. Moreover, they might become infected through mechanical transmission in pixels neighboring tsetse infested pixels [42], [43]. A longitudinal monitoring of sentinel herds in the target area showed that herds located in infested pixels had annual trypanosomosis incidences of 80–90% whereas those located in the adjacent pixels had incidences of 20–30% (data not shown); these data confirm that the infection risk was 3–4 times higher in the tsetse-infested areas as compared to adjacent areas. Even with a reduced incidence, the production capacity of improved farming systems measured in this study is probably still lower than it would be in the absence of the disease [11]. One of the main limitations of our study is that production costs associated to each farming system could not be assessed and as a result we estimated the increase of cattle sales rather than its benefits. Moreover, in extensive systems, cash is not the only component of livestock output, with home slaughter, gifts and transfers in and out of the herd. However, the potential reduction of the total cattle population by 45% might balance the increase of production costs associated to improved milk systems [10]. For improved meat systems, the production practices (and thus costs) are very similar to the traditional one. Moreover, the reduction of the herd size will have unquantifiable positive impacts on the environment in the target area which suffered from overgrazing and competition for food that occurred between cattle, horses that are used for animal plowing and transport, and sheep that are present in large numbers. During the socio-economic surveys described above, we observed 8,048 goats, 1,469 sheep, 276 donkeys and 206 horses in the farms surveyed. Small ruminants must also be taken into consideration in the competition for food, but equines can be omitted in this context. However, the diet of small ruminants includes more ligneous species (trees and shrubs) as compared to that of cattle and horses thus partially compensating the impact of overgrazing caused by cattle [44].

Another limitation of the study is that we did not differentiate between cattle sales resulting from increased productivity of the herds and those resulting from commercial activities (calves bought, fattened and sold). This is particularly true for improved meat systems where this activity is common which probably lead to an underestimation of the calving rate in this group. We however consider that it is still a type of intensification, since this type of cattle is generally maintained in zero-grazing units and the animals are sold younger than in the traditional system (around three years).

Before the implementation of the elimination programme, the maintenance of exotic cattle (Holstein, Jersey, Montbeliard, etc) in zero-grazing units was only possible by keeping these trypano-susceptible animals under constant prophylactic treatment with trypanocidal drugs, which is very costly and difficult to sustain in the long term [45]. The present study showed that farmers with an improved milk system had higher costs than farmers in the other systems, but the costs were still very low in comparison to the value of the cattle. It is likely that the costs as declared by the farmers in the present socio-economic study were underestimated because state veterinary staff conducted the surveys and farmers were reluctant to admit that they mainly rely on private veterinarians or treat their animals on their own [39]. Improved milk systems were however almost all located outside the tsetse infested pixels and the only two farms located inside the tsetse area declared no sales (cattle or milk) in 2010. Although there are big industrial farms that succeeded in securing networks for selling important daily volumes either by developing their own distribution chain or selling their produce to industrial local manufacturers that produce and market packed milk like Kirène, these have remained a minority. The best milk production observed during the survey was 1400 L per cow per lactation. Considering that a dairy cow of an improved breed can produce 5000 to 10,000 L milk/lactation, there is still much room for improvement.

Like in the remainder of West-Africa most cattle in the Niayes area are grazing on fallow land and natural pastures and this can be considered as an ecosystem service. These pastures tend to disappear due to competition for space with crops and artificial surfaces (buildings, roads,…) and the remaining protected areas in the Niayes are suffering severely from overgrazing. Overgrazing impacts the health of an ecosystem (land degradation) [3] and reduces the carrying capacity of natural pastures. Moreover, the access to crop residues becomes more and more difficult, in relation to an increasing competition between traditional and modern famers with different modalities of negotiation with the crop farmers (trade of cattle manure, purchase). A few farms that practice intensive livestock keeping have developed alternative strategies to improve their productivity like the farming of cereals (and their conservation by ensilage), procurement of cattle-cake and beer waste products. Most of the inputs, especially groundnuts tops, are imported from other areas of Senegal, or even from Mali. The agro-industrial waste-products include rice bran, brewer's spent grain, groundnuts shells, treacle, groundnuts and cotton cattle-cake. Some factories also produce specific cattle food but they are expensive. Even if the rotation of forage and crops is a good way to improve soil fertility, most of the space is used for crops due to the competition between animal and human food [10].

### Conclusion

The tsetse elimination project in the Niayes is considered by the Government of Senegal as an ecologically sound strategy to intensify cattle production that will result in a decrease in cattle density, and the use of more productive cattle in urban areas. The data of this study have indicated that the elimination of the *G. p. gambiensis* population from the Niayes will bring major overall socio-economic benefits for the farmer community that is composed of several farming systems, more or less exposed to the disease. However, the adoption of new technologies is generally difficult because it requires changes of the socio-technical regime including social norms and associated practices, for example the management of exotic breeds [46], [47]. In the case of the improved meat system, practices and social norms are similar to the traditional system whereas in the case of improved milk systems, complete technological packages and associated norms must be changed (for example the attractiveness of local milk for consumers in comparison to powdered milk). We are presently studying individual and collective trajectories of herders based on comprehensive analyses to better estimate the mutation rates [48]. Moreover, the benefits will have to be evaluated more accurately using ex-post socio-economic surveys.

## Supporting Information

Supporting Information S1 Data used in the socio-economic analysis.
(XLSX)
